# Optical coherence tomographic angiography detects retinal vascular changes associated with pituitary adenoma

**DOI:** 10.1016/j.ajoc.2022.101711

**Published:** 2022-09-15

**Authors:** Ping Wei, Julie Falardeau, Aiyin Chen, Jie Wang, Liang Liu, Yali Jia, David Huang

**Affiliations:** Casey Eye Institute and Department of Ophthalmology, Oregon Health and Science University, Portland, OR, USA

**Keywords:** Pituitary adenoma, OCT angiography, Bitemporal hemianopia

## Abstract

**Purpose:**

To report the distinct pattern of retinal perfusion loss captured on optical coherence tomographic angiography (OCTA) in a case of compressive optic neuropathy associated with pituitary adenoma.

**Observations:**

A 51-year-old male had bitemporal hemianopia caused by a pituitary adenoma that compressed the optic chiasm. OCTA scans in both eyes showed peripapillary nerve fiber layer plexus defects in the nasal hemispheres and papillomacular corridors. On macular scans, the ganglion cell layer plexus showed papillomacular defects. The perfusion defects corresponded with thinning on structural OCT measurement and loss of sensitivity on visual field tests.

**Conclusions and importance:**

Chiasm compression produces a characteristic pattern of perfusion loss that can be recognized OCTA. This knowledge may be useful in the diagnosis and classification of optic neuropathies.

## 1 Introduction

Pituitary adenoma accounts for 10–15% of all intracranial tumors.^1^ It can produce midline compression of the optic chiasm from its superior aspect, causing bitemporal visual field (VF) loss.^2^^,^^3^ This compressive optic neuropathy exhibits the characteristic “band or bowtie atrophy” of the optic nerve head, damaging axons originating in the nasal hemiretina, with axonal loss affecting predominantly the nasal and temporal quadrants of the optic disc.^4^ Macular retinal neural loss tends to affect the nasal hemisphere and leave the temporal side relatively preserved.^5^

These damages can be evaluated by optical coherence tomography (OCT), which show characteristic patterns of thinning of the peripapillary retinal nerve fiber layer (NFL) and macular ganglion cell complex (GCC).^5^, ^6^, ^7^, ^8^ OCT angiography (OCTA)^9^ is a new imaging modality that can also be used to evaluate optic neuropathies, with possible advantages over structural OCT such as earlier and better correlation with VF loss.^10^^,^^11^ OCTA changes in compressive optic neuropathy have been reported in two previous articles.^12^^,^^13^ In this report, we use quantitative vessel density analysis^14^ to identify the pattern and severity of perfusion loss on peripapillary and macular OCTA in a case of compressive optic neuropathy due to pituitary adenoma. The assessment is based on the premise that since OCTA signal is related to blood cell velocity,^15^ Loss of observable capillaries and larger vessels on OCTA indicates a loss of perfusion. A projection-resolved OCTA algorithm^16^^,^^17^ is used to accurately map the macular ganglion cell layer plexus (GCLP)^18^ to identify local ganglion cell perfusion loss.

## 2 Case report

**Fig. 1:**
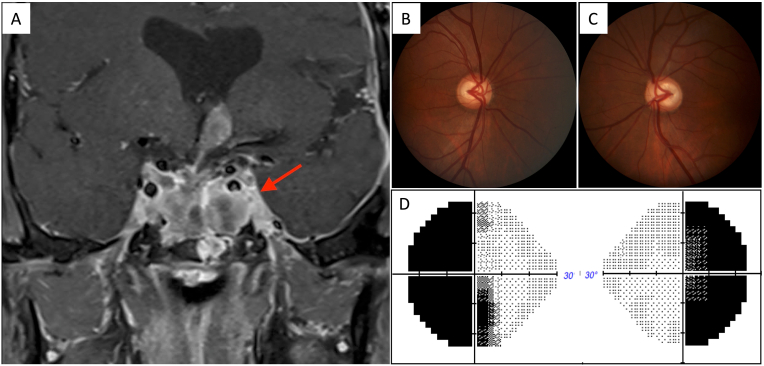
A: Magnetic resonance imaging revealed a heterogeneous mass (red arrow) in the sellar and suprasellar regions, as well as bilateral cavernous sinus. Right (B) and left (C) fundus photographs showed cup-to-disc ratio of 0.6 in the right eye and 0.7 in the left eye with mild pallor nasally and temporally in both eyes. Humphrey 24–2 visual fields (D) showed a bitemporal hemianopia. (For interpretation of the references to colour in this figure legend, the reader is referred to the Web version of this article.)

The patient was a 51-year-old male who was diagnosed with pituitary adenoma in 2001. He underwent five tumor resections, with the last 7 years ago (in 2001, 2002, 2011, 2011 and 2012). The histology of resected tumor showed loss of the normal acinar architecture, demonstrated on hematoxylin and eosin and reticulin stain, and was diagnosed pituitary adenoma. The tumor is negative for ACTH, growth hormone and prolactin by immunohistochemistry. The last resection was followed by radiation therapy. However, one year after the final resection, the tumor recurred, invading the cavernous sinuses and the third ventricle. Then he started chemotherapy with temozolamide, which arrested the tumor growth. The most recent magnetic resonance imaging (MRI) revealed a heterogeneously enhancing mass in the sellar and suprasellar regions (Fig. 1). The patient had no other history of ocular or systemic diseases. Radiation retinopathy was excluded since the retinal examination was entirely normal.

The Snellen visual acuity was 20/20 and 20/30, and the intraocular pressure was 8 and 9 mmHg in the right and left eyes, respectively. Fundus photographs showed enlarged cup-to-disc ratio and segmental rim pallor in both eyes (Fig. 1). Humphrey 24-2 VF test (Humphrey Field Analyzer II, Carl Zeiss, Inc.) showed a bitemporal hemianopia (Fig. 1). The mean deviation was −13.9 dB and −17.9 dB, and pattern standard deviation was 16.1 dB and 16.6 dB in the right and left eyes, respectively. The VF and visual acuity have been stable for the past 18 years and has not changed in the course of surgical, radiation, and medical treatments.

## 3 Material and methods

OCT and OCTA imaging were performed using a 70-kHz 840-nm wavelength spectral-domain OCT system (Avanti, Optovue, Inc., Fremont, California, USA), running the AngioVue OCTA software. The 4.5x4.5-mm disc and 6x6-mm macular OCTA scans were obtained from both eyes. The OCTA scans were processed using a custom software, the COOL-ART,^19^ which removed flow projection artifacts^17^ and calculated reflectance-compensated^20^ vessel density (VD) and capillary density (CD). The VD maps were obtained by computing the fraction of area occupied by flow pixels after low-pass filtering 41 × 41-pixel elements in each slab. Arterioles and venules (larger vessels) were automatically identified by thresholding the *en face* mean projection of OCT reflectance within the all-plexus slab. Due to the darker shadow cast by larger vessels, relative to capillaries, the average reflectance of the retina is reduced at the location of large vessels. After these larger vessels were excluded, the remaining angiogram was used to compute CD. The structural thickness metrics were extracted from the standard OCT scans. Normative OCTA data was obtained from 30 healthy eyes of 30 subjects.^21^

## 4 Results

**Fig. 2:**
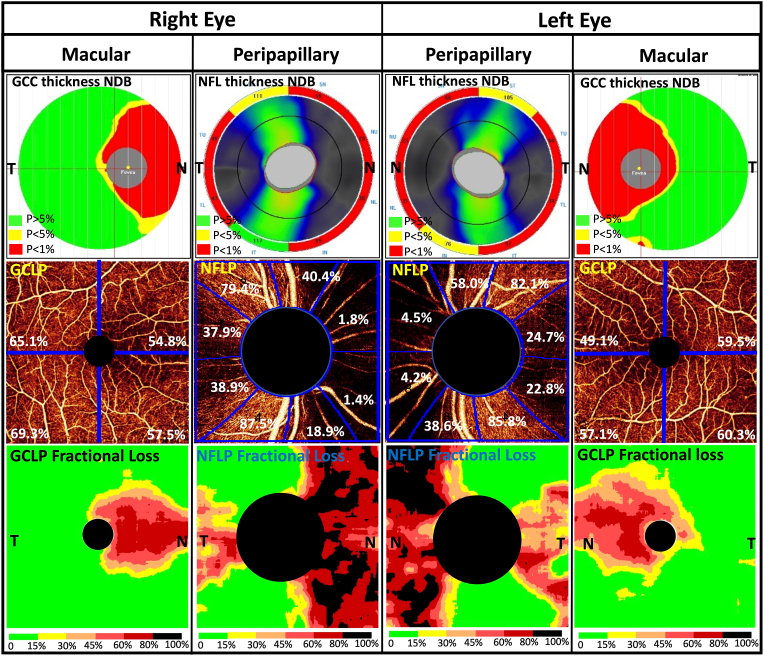
Standard structural OCT normative classification maps (top row) show that sectoral macular ganglion cell complex (GCC, 6-mm map) thinning and peripapillary nerve fiber layer (NFL, 5-mm map) thinning (red and yellow areas) compared to the normative database (NDB). The en face OCTA (middle row) show macular ganglion cell layer plexus (GCLP) vessel density (% area values shown on images) was reduced along the papillomacular bundle, and peripapillary nerve fiber layer plexus (NFLP) capillary density (% area values shown) was reduced in the nasal hemisphere and temporal quadrant. The perfusion defects are shown even more clearly on the fractional loss maps (bottom row). (For interpretation of the references to colour in this figure legend, the reader is referred to the Web version of this article.)

Peripapillary OCT showed severe NFL thinning in the nasal hemisphere and temporal quadrant in both eyes (Fig. 2). The superotemporal and inferotemporal NFL bundles were also affected, but to a lesser degree. This was matched on OCTA by nerve fiber layer plexus (NFLP) perfusion loss in the nasal hemisphere and temporal quadrant as demonstrated by both sectoral CD and fractional perfusion loss maps (Fig. 2).

Macular OCT showed GCC thinning in the papillomacular corridor (nasal quadrant) in both eyes. This was matched on OCTA by ganglion cell layer plexus (GCLP) VD loss. It is notable that the GCLP VD loss crossed the vertical midline into the superotemporal sector of the fovea in the left eye but not the right eye. This correlated with the decreased visual acuity and small inferonasal extension of scotoma in the left eye. We also analyzed the VD in the intermediate capillary plexus (ICP) and deep capillary plexus (DCP) and found they were within normal limits relative to previously published values.^21^

## 5 Discussion

This case report shows that the structural thinning on OCT and perfusion defects on OCTA generally match the bitemporal hemianopia pattern on VF in this patient with chiasmal compression from a pituitary adenoma. These defects occurred in the ganglion cell related layers as would be expected for an optic neuropathy, and spares the deeper retinal layers and plexuses.^21^, ^22^, ^23^ These thinning pattern have been observed by several previous OCT studies.^5^^,^^8^^,^^24^

The pattern of peripapillary NFLP perfusion loss we found was identical to those found by Dallorto et al. and Suzuki et al. group.^12^^,^^13^ Thus the pattern of nasal hemispheric and temporal quadrantic NFL/NFLP defect can be considered characteristic for chiasmal compression. This OCT pattern is in agreement with the known bowtie patterns of optic disc pallor and bitemporal hemianopia pattern on VF.

For macular OCTA, our method of analysis is different from the previous two reports,^12^^,^^13^ which showed OCTA of the superficial vascular complex (SVC), a combination of both the NFLP and GCLP. In this report, we used a projection-resolved OCTA algorithm to remove flow projection artifacts,^17^ which allowed us to measure the GCLP without the interference of projection from the overlying NFLP. Furthermore, we applied fractional loss map to detect the pattern of loss relative to a normative database. We anticipated that this would allow us to observe the loss of GCLP in the entire nasal halves of both maculas to match the bitemporal hemianopia pattern, which implies binasal hemispheric pattern of ganglion cell loss.^5^ Instead, we found that GCLP was significantly reduced only along the papillomacular bundle, and the superotemporal and inferotemporal sectors were largely spared. An explanation of this discrepancy is that the GCLP partially supplies the overlying NFL along the thick superior and inferior arcuate nerve fiber bundles. Along the papillomacular bundle, the NFLP and GCLP were only moderately attenuated, in contrast to the deep VF defect. A possible explanation is that part of the NFLP and GCLP serves as a conduit between the larger retinal vessels and the deeper retinal plexuses (ICP and DCP), which are denser in the macula (compared to the periphery) and relatively well preserved in optic nerve diseases. In comparison to these milder macular changes, the loss of NFLP in the nasal half of the peripapillary region was more complete. This case also suggests that central visual acuity may be affected when foveal GCLP loss crosses the vertical midline.

## 6 Conclusions

In this case of chiasmal compression caused by a pituitary macroadenoma, OCTA detected characteristic patterns of vascular loss in the peripapillary and macular regions, which was in general consistent with the anatomic structure of the chiasm, visual field defects and structural OCT changes. OCTA may be uniquely useful in situations where the patient is unable to produce reliable visual function tests, and hypothetically in acute disease where retrobulbar degeneration has not caused retinal structural loss yet.

## Funding sources

Supported by 10.13039/100000002NIH grants R01 EY023285, R01 EY010145, P30 EY010572, by unrestricted departmental funding from Research to Prevent Blindness (New York, NY). The sponsor or funding organization had no role in the design or conduct of this research.

## Financial Support

The study was supported by 10.13039/100000002NIH grants R01 EY023285, P30 EY010572, R01 EY010145 from the 10.13039/100000002National Institutes of Health, Oregon Health & Science University (OHSU) foundation, and an unrestricted grant from 10.13039/100001818Research to Prevent Blindness.

## Authorship

All authors attest that they meet the current ICMJE criteria for authorship.

## Author contributions

Ping Wei: Data curation, Conceptualization, Writing- original draft preparation, Julie Falardeau: Resources, Aiyin Chen: Writing-review & editing, Jie Wang: Software, Liang Liu: Methodology, Yali Jia: Supervision, David Huang: Supervision, Writing- Reviewing & Editing.

## Patient consent

Consent for publication was received from the individual whose data is presented in this case report.
